# Impacted Permanent Mandibular Canines: Epidemiological Evaluation

**DOI:** 10.3390/jcm12165375

**Published:** 2023-08-18

**Authors:** Eanda Agastra, Margherita Saettone, Simone Parrini, Giovanni Cugliari, Andrea Deregibus, Tommaso Castroflorio

**Affiliations:** 1 Department of Surgical Sciences, C.I.R. Dental School, University of Turin, Via Nizza 230, 10126 Turin, Italy; meme9595@hotmail.it (M.S.); simo.parrini@gmail.com (S.P.); andrea.deregibus@unito.it (A.D.); tommaso.castroflorio@gmail.com (T.C.); 2 Department of Mechanical and Aerospace Engineering (DIMEAS), Politecnico di Torino, Corso Duca degli Abruzzi 24, 10129 Turin, Italy; 3 Department of Medical Sciences, University of Turin, Corso Dogliotti 14, 10126 Turin, Italy; cugliarigiovanni@gmail.com

**Keywords:** orthodontics, mandibular canines, tooth impaction, transmigration, epidemiology

## Abstract

Background: The aim of this study was to evaluate the epidemiology of impacted and transmigrated mandibular canines in a large orthodontic population referred to the University of Turin. Methods: Panoramic radiographs, intraoral photographs, and dental casts of 2119 patients referred to the Department of Orthodontics at the University of Turin, Italy, between 1995 and 2022 were reviewed. These patients were divided into two groups. Group A included 1479 patients found in the Dental School archive before 2017, more specifically between 1995 and 2017. These patients were examined in order to calculate the prevalence of impacted and transmigrated mandibular canines. From 2017 to 2022, the records of 640 new patients were examined (GROUP B) in order to calculate the incidence of these occurrences. Results: The prevalence of mandibular canine impaction in Group A was found to be 1.7%, with a total of 25 patients having mandibular canine impaction. A prevalence of 0.3% was found for mandibular canine transmigration (Group A). The incidence of mandibular canine impaction was found to be 2%, with a total of 13 patients with mandibular canine impaction (Group B). Mandibular canine transmigration was found in 1 of 640 participants (Group B). Conclusions: Twenty-five of 1479 patients had impacted mandibular canines, resulting in a prevalence value of 1.7%. The incidence was found to be 2%, with 13 of 640 patients having impacted mandibular canines. These results show higher prevalence and incidence rates of mandibular canine impaction when compared with previously published data.

## 1. Introduction

Dental anomalies, such as alterations in number, sequence of tooth eruption, tooth structure, tooth size, and lack of eruption, often present a significant challenge for dentists. Many of these undiagnosed and untreated anomalies can cause significant problems in areas such as orthodontics, pediatric dentistry, prosthodontics, restorative dentistry, and endodontics [1].

Failure of tooth eruption into the oral cavity because of either insufficient space or the presence of an entity blocking its path of eruption may lead to different conditions such as impaction, transmigration, retention, or malposition/displacement [2]. Tooth impaction is a condition defined by the failure of a tooth to erupt into the dental arch within the physiological and time limits of normal tooth eruption [3,4,5]. Other authors define it as the absence of the tooth in the dental arch after the complete development of its root, or at least six months after the eruption of its contralateral tooth [6]. Abron et al. describe tooth impaction as “a delay or a block of eruption” [4]. Impacted teeth have a considerable delay in eruption time, or they are expected not to erupt based on the clinical and radiographic evidence [5]. It is important to emphasize the presence of various general or local causes of delayed eruption of mandibular canines, such as mechanical obstacles such as bone tissue or adjacent tooth elements, etc., that block the eruption of the tooth and make it get stuck inside the alveolar process, lack of space, traumatic factors, and genetic disorders [3,5,7,8,9,10].

An abnormal intraosseous pre-eruptive migration of the unerupted tooth from its physiological position across the midline is termed transmigration [2]. Transmigration is present when part of the length or at least half of the length of the crown of the impacted tooth crosses the midline. It is a very rare eruption disorder, typical of impacted teeth, and it is commonly seen in the permanent dentition of the mandibular arch and only occasionally in the maxilla. The most common tooth affected is the mandibular canine [11]. This tooth migration usually begins during the mixed dentition stage and may go on for many years. Since almost all canine transmigrations are asymptomatic, except in cases where patient notices the loss of aesthetics due to the absence of the transmigrated canine, they are usually discovered during routine examinations [1,2,11,12,13,14,15].

The canines play a very important functional and aesthetic role in human dentition, and altered eruption of these teeth is a major patient concern [11]. The presence of canines in dental arches is also related to canine guidance [16].

Due to the eruption pattern and sequence, canines are prone to impaction. Maxillary canines are the second most frequently impacted teeth after third molars. Maxillary canines are affected 20 times more frequently than mandibular canines. Normally, in the mandible, the eruption sequence of the permanent dentition follows an anterior-to-posterior pattern. In the maxilla, usually the eruption of premolars follows the incisors, after which the canines are expected to erupt into the dental arch at about 11–12 years of age. The maxillary canines normally develop high in the upper jaw. They are among the last teeth to erupt (except the third molars) and must travel a considerable/longer distance from their point of development to normal occlusion. Consequently, there is an increased potential for mechanical interference leading to displacement and subsequent impaction of the canines. Another reason leading to a more frequent impaction of maxillary canines could be the limited space for eruption, as maxillary canines erupt between teeth that are already in occlusion. The second molar may further reduce the space [2].

Regarding mandibular canines, it is normal for the permanent canines to erupt labially from the primary canines. This labial eruption results in an increase in the intercanine distance, which creates more space in the mandibular arch. Therefore, in cases of normal eruption, a buccal bulge produced by the mandibular canine should be palpable prior to eruption at any time from the dental age of 9 years. The mandibular canines normally erupt before the maxillary canines. The most favorable sequence of eruption of mandibular teeth is: first molar, central incisor, lateral incisor, canine, first premolar, second premolar, second molar, and third molar. If the sequence of eruption is favorable, the canines should erupt prior to the premolars. The permanent mandibular canines usually erupt at about 10–11 years of age [9].

Impacted mandibular canines lose their ability to erupt due to different positions and angulations in the lower jaw [9]. The prevalence of mandibular canine impaction has been reported to be 0.92–1.35% [17]. Furthermore, the prevalence of mandibular canine transmigration has been found to be 0.1–0.76% [1,2,11,13,18].

However, the epidemiology of these conditions in Western countries is not yet well known.

Even though the most impacted teeth do not have symptoms, some of them can cause complicated situations such as lingual or labial positioning of the impacted tooth, migration of adjacent teeth, and eventually loss of arch length, external root resorption, and marginal bone resorption adjacent to the impacted tooth and the neighboring teeth, cysts, tumors, resorption of adjacent teeth, infections, pain, and aesthetic problems due to the malposition of the anterior mandibular teeth [1]. Therefore, the orthodontic solution for impacted and transmigrated mandibular canines could be challenging. Knowledge of the frequency of these dental anomalies is essential for appropriate interceptive treatment, management of complications, and treatment planning. 

Mandibular canine impaction and transmigration are rare conditions, and there are very few studies concerning these occurrences. Consequently, it is difficult for the dental practitioner to find reliable data concerning epidemiological features and diagnostic protocols for these events. 

In order to overcome these epidemiologic limitations, the aim of the present study was to estimate the prevalence and incidence of impacted and transmigrated mandibular canines in a large sample of the orthodontic population referred to the University of Turin between 1995 and 2022.

## 2. Materials and Methods

Panoramic radiographs, dental casts, and intraoral photographs of a total of 2119 patients referred to the Clinical Department of Orthodontics of the Dental School at the University of Turin, Italy, between 1995 and 2022, were reviewed in order to detect subjects with impacted and transmigrated mandibular canine teeth and consequently to measure the frequency of these conditions. The present study was initiated in 2017. These patients were divided into two groups. 

The first group (Group A) included the patients who were examined before 2017, and more specifically in the period from 1995 to 2017. In Group A, the records of 1479 patients [682 (46.11%) males and 797 (53.89%) females] were examined. These records were found in the archives of the Dental School. Group A patient records were examined to calculate the prevalence of canine impaction and transmigration in 2017. 

The second group (Group B) included patients examined after 2017. In Group B, the records of 640 new patients [290 males (45.3%) and 350 (54.7%) females] were examined during the period from 2017 to 2022. These records were reviewed in order to calculate the incidence of these conditions over this period of time. 

All these data are presented in Table 1.

The age of these patients ranged from 12 years and 11 months to 47 years and 8 months, with a mean age of 14.07 years. Some of the patients with mandibular canine impaction had their first orthodontic visit at about 9 years and 10 months of age. After their first visit, they did not come in for routine checkups until they were 14 years or older, the age at which they were diagnosed with mandibular canine impaction. A comprehensive chart review was conducted for all participants.

The following clinical signs were indicative of canine impaction [1]:

- Delayed eruption of the permanent canine and prolonged retention of the deciduous canine
- Delayed eruption, distal tipping, or migration of the lateral incisor.
- Proinclination of mandibular incisors

The number, positions, and locations (right or left) of impacted/transmigrated mandibular canines, as well as patient gender, age, retained deciduous canines, and any other associated pathologies, were noted after evaluation of the patient’s general history and clinical and radiographic records.

**Figure 1:**
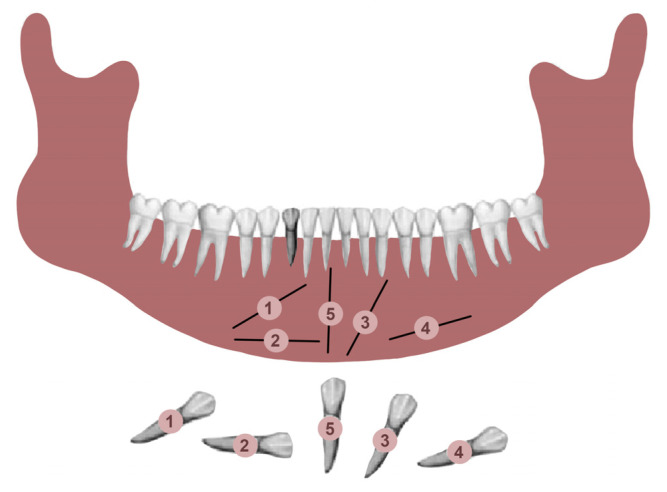
Diagrammatic representation of the 5 patterns of transmigration, with a retained deciduous right mandibular canine according Mupparapu.

Transmigrated mandibular canines were classified according to Mupparapu’s classification [19]. Mupparapu used five criteria to classify the transmigrated mandibular canines into five types. This classification was based on their migratory pattern, the inclination of the longitudinal axis of the canine, the final position inside the mandible, and the relation of the canine crown with adjacent teeth, the midline, and the contralateral erupted canine (Figure 1) [19].

Type 1: the canine is positioned mesioangularly across the midline, labial or lingual to the anterior teeth, and the crown portion crosses the midline.

Type 2: the canine is impacted horizontally near the inferior border of the mandible below the apices of the incisors.

Type 3: the canine has erupted either mesially or distally to the opposite canine.

Type 4: the canine is horizontally impacted near the inferior border of the mandible below the apices of either premolars or molars on the opposite side.

Type 5: the canine is positioned vertically in the midline, with the long axis of the tooth crossing the midline. 

Given the retrospective nature of the study and the use of exclusively radiographic exams, dental casts, and intraoral photographs, no informed consent was considered necessary. 

### Statistical Analysis

A statistical analysis on the prevalence and incidence samples was performed. Prevalence and incidence were calculated as P = (n cases/n patients in the study) × 1000. 

The normality assumption of the data was evaluated with the Shapiro-Wilk test; homoscedasticity and autocorrelation of the variables were assessed using the Breusch-Pagan and Durbin-Watson tests. 

## 3. Results

A total of 38 out of 2119 patients were found to have mandibular canine impaction, while 5 of 2119 participants were found to have mandibular canine transmigration. These values are shown in Table 2.

**Figure 2:**
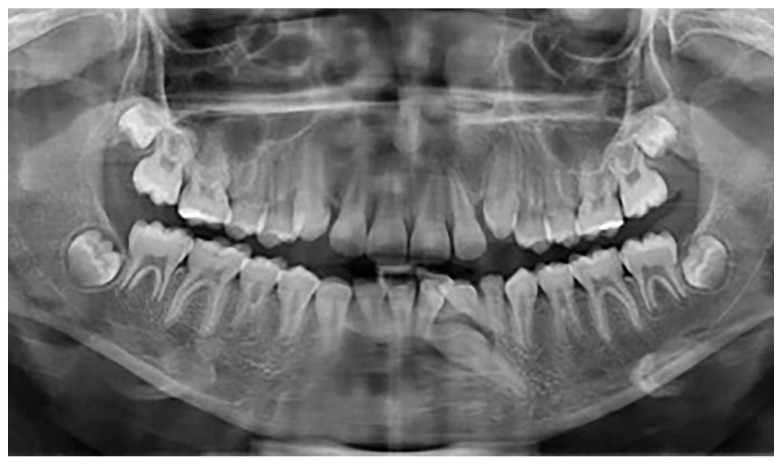
Mandibular impaction of left canine and presence of a supernumerary tooth.

A total of 25 patients with mandibular canine impaction were found in Group A. Of these 25 patients, 14 were male and 11 were female. Canine impaction was bilateral in 12 patients and unilateral in 13 patients, with a total of 37 impacted permanent mandibular canine teeth. 21 impacted canines were found on the left side, and 16 canines on the right. 16 patients (64%) had retained deciduous canines at the time of diagnosis. In three cases, the impacted canines were transposed in the lateral incisor region. In one of the patients, there was a supernumerary tooth in the lateral incisor region, and the primary canine was still present in the dental arch (Figure 2). In one patient in whom the primary canine and primary lateral incisor were retained, impaction of both the lower permanent canine and lower permanent lateral incisor was noted. One patient was found to have complete impaction of all four canines. None of the patients had traumatic episodes, and none of them had systemic disorders. All the patients were asymptomatic. 

**Figure 3:**
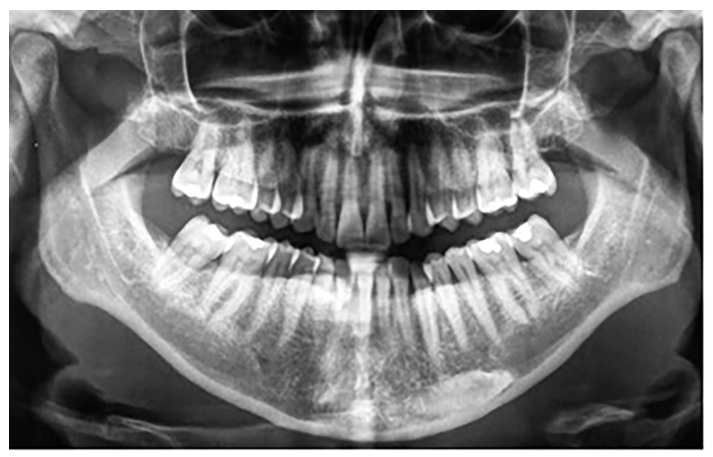
Patient with a Type 4 transmigrated right mandibular canine.

Besides these 25 patients with mandibular canine impaction, 4 of 1479 participants, three females and one male, were found to have mandibular canine transmigration. Considering the small size of the sample, no gender differences were observed. All patients had retained primary canines. In all four cases, the transmigrated canines were unilateral and impacted, with three involving the right side and one on the left side. Two canines were associated with dentigerous cysts. Of the four transmigrated mandibular canines in the present study, one was classified as type 1 and three were classified as type 4 according to Mupparapu’s classification (Figure 3). The summary of these findings is shown in Table 3.

The prevalence values of impacted permanent mandibular canines and transmigrated mandibular canines retrieved from Group A are reported in Table 4. 

**Figure 4:**
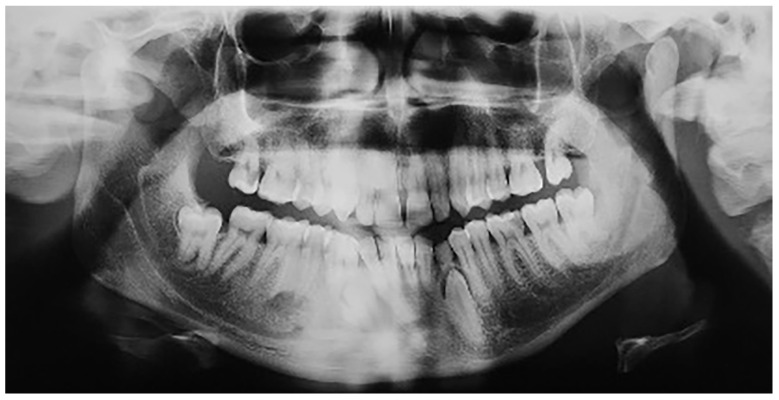
Patient with impaction of mandibular canines and retained deciduous canines.

In group B, a total of 13 patients were found to have mandibular canine impaction. Impaction of the canines was bilateral in 1 patient and unilateral in 12 patients, for a total of 14 impacted permanent mandibular canine teeth. Four patients still had retained deciduous canines at the time of diagnosis (Figure 4). In two cases, the impacted canines were transposed in the region of the lateral incisors. Besides these 13 patients with impacted mandibular canines, one (female) of the 640 participants was found to have mandibular canine transmigration. The transmigration was unilateral. In this patient, the primary canine was retained. None of the patients had traumatic episodes, and none of them had systemic disorders. All the patients were asymptomatic. 

The summary of these findings is shown in Table 5. 

The incidence values of permanent impacted and transmigrated mandibular canines calculated from Group B are reported in Table 6.

## 4. Discussion

Different frequencies were found among different ethnic groups. These different ethnic samples may result in higher or lower rates for some anomalies. Dental practitioners who are aware of ethnic differences in the occurrence of these dental anomalies are more likely to recognize them during routine examinations and to predict normal patterns of tooth development and eruption, allowing efficient clinical intervention to avoid complications [20]. However, failure of eruption of permanent mandibular canines is a very rare phenomenon, and there are very few studies in the literature concerning the frequency of occurrence of impacted mandibular canines [14,21]. According to the literature, the prevalence of impacted mandibular canine teeth ranges from 0.07% to 1.36% among different populations. Grover and Lorton found a prevalence of 0.22%, and similar results were confirmed by Chu et al. with a prevalence value of 0.07% [22,23]. In another Chinese population, a prevalence value of 0.3% was found [3]. In a Turkish population, Aydin et al. found 20 patients with mandibular canine impaction with an overall prevalence of 0.44%, while Topkara and Sari found a value of 0.92% [14,24]. A higher value of 1.36% was found in a study conducted at Lagos Teaching Hospital, Nigeria [25].

In the present study, a prevalence value of 1.7% was found. This value is higher than previously published literature. 

Sanu and Adeyemi studied 1250 patients from January 2001 to September 2008 and found an incidence value of 1.36%, with 17 patients having impaction of mandibular canines [25]. Grover and Lorton found an incidence of 0.3%, with only 11 impacted mandibular canines in the 5000 patients studied [22]. Yavuz found 65 impacted mandibular canines in a Turkish population of 5022 patients, with an incidence of 1.29% [5]. 

The incidence of impacted mandibular canines in the present study was found to be higher than published data, with a value of 2%. 

In this study, mandibular canine transmigration was also evaluated. Although the impaction of maxillary canines is more frequent than that of mandibular canines, transmigration is less frequent in the maxilla because of anatomical conditions. The anterior mandibular region has a large cross-sectional area and may predispose to a higher frequency of transmigration of mandibular canines [14,26]. However, this larger cross-sectional area was considered by Joshi to be a consequence rather than a cause of the transmigration of canines [27]. It has been reported that most of the pre-eruptive migration distance is observed in the early stages of tooth development when root development is not yet complete [28]. However, even after root development is complete, this migration process may continue for many years and is thought to follow the path that offers less resistance [29,30].

Previous studies reported a low value for the frequency of transmigration, ranging from 0.1% to 0.76%. In this study, a prevalence of 0.3% was found, with 4 out of 1479 participants having transmigration of mandibular canines. Shah et al. found a prevalence of 0.1% in 7886 individuals [31]. Aktan et al. found a frequency of 0.34% in a Turkish population of 5000 patients, while another study found a prevalence of 0.11% in a Greek population of 3586 patients [2,15]. An Indian study found a frequency of 0.19% in 3500 patients [26]. A higher frequency of 0.76% in 6840 patients was found in a more recent study in a Spanish orthodontic population [18]. However, in studies such as Zvolanek’s, no cases were found in 4000 individuals [32]. Similarly, Fardi et al. found no transmigrated mandibular canines in a Greek population of 1239 patients [33]. Our results are consistent with most of the previous studies. 

These discrepancies among studies concerning the frequency of the occurrence of mandibular canine impaction could be due to methodological differences. Different populations, ranging from orthodontic patient populations to the general population, different sample sizes, and different racial groups, have been studied. In addition, the current data derive mainly from specific populations, such as Chinese and Turkish, and therefore may not be fully representative of the general Caucasian population. Consequently, new studies on more representative populations are needed in the future [17].

The present study was carried out in the Dental School of the University of Turin, in the Department of Orthodontics. The results are expected to be representative of an important part of the population of the Piedmont Region in Italy.

In the published literature, there are few studies concerning the association between the frequency of mandibular canine impaction and transmigration and gender and side of localization. Two different studies conducted by Aydin et al. and one study conducted by Sanu et al. reported more female than male patients with impacted mandibular canines [2,14,25]. On the contrary, the present study found more male patients (14 male) than female patients (11 female), in accordance with the systematic review of 2016 [17]. 

More impacted canines located on the right side than on the left side were found in the study of Yavuz et al. and in the study of Sajnani and King [3,5]. Unlike these studies, the present study found more impacted mandibular canines located on the left side of the jaw than on the right side (21 impacted canines on the left side and 16 canines on the right side).

Although the existing literature reported a prevalence of unilateral canine impaction, in this study, the distribution of bilateral and unilateral impactions was almost equivalent (12 bilateral and 13 unilateral) [3,5,14]. For mandibular canine transmigration, it is more common to have unilateral mandibular canine pre-eruptive intraosseous migration, and in the present study, all the patients had unilateral mandibular canine transmigration. However, the published literature has also reported a few cases of bilateral canine transmigration.

According to a literature review performed by Mupparapu, Type 1 of transmigrated mandibular canines was the most common pattern. This was followed by Type 2, Type 4, Type 3, and Type 5 in decreasing order [19]. However, different studies have reported different results. In the present study, one canine of Type 1 and three canines of Type 4 were found, which is in contradiction with the literature. These differences among studies could be due to the small number of transmigrated mandibular canines found. 

The simplest interceptive procedure to prevent impaction of the permanent canines is the timely extraction of the primary canines. This procedure usually allows the permanent canines to upright and erupt properly into the dental arch [34,35,36]. In the patients of the present study (Group A), 16 (64%) patients had retention of the primary canines. These patients did not undergo timely extraction of the primary canines. Therefore, ineffective interceptive treatment may have contributed to the higher frequency of permanent canine impaction in this study. In other studies, three out of 64 patients (4.6%) and 12 out of 65 patients (18.5%) had retained primary canines [3,5]. These data may suggest that the frequency of mandibular canine impaction was found to be lower in these studies due to more efficient and timely interceptive treatment. 

In general, impacted teeth are asymptomatic. Nevertheless, in the literature, symptomatic cases with chronic infection, swelling, and pain have been reported [5,21,23]. In the present study, all patients were asymptomatic.

However, this study has some limitations, as the sample population was representative of the patient pool at the Dental School of Turin. Wider population groups should be studied in Italy and other Western countries. Nevertheless, the prevalence and incidence rates of mandibular canine impaction found in this study may reflect the prevalence and incidence rates of these anomalies in the general population. Another limitation of this study could be the possible presence of artifacts in Dental Panoramic Tomography and, therefore, misinterpretation in the anterior region of the mandible. Possible causes of artifacts include superimposition of intervertebral spaces, depression in the mental region of the mandible, superimposition of radio-opaque structures, reduced image detail compared with intraoral views, and uneven magnification. For these reasons, Dental Panoramic Tomography could be inappropriate for imaging impacted mandibular canines [37]. 

## 5. Conclusions

The prevalence of mandibular canine impaction was found to be 1.7%, with 25 of 1479 patients having impacted mandibular canines, and the incidence was found to be 2%, with 13 of 640 patients having impacted mandibular canines. These results show higher prevalence and incidence rates of mandibular canine impaction when compared with previously published data.

## Figures and Tables

**Table 1:** Number and gender of patients examined in different periods of time.

	Year 1995–2017 (Group A)	Year 2017–2022 (Group B)	Year 1995–2022 (Group A + B)
Total number of patients	1479	640	2119
Females	797	350	1147
Males	682	290	972

**Table 2:** Prevalence of Impacted and Transmigrated permanent canines in the Cohort Study (n = 2119).

	Impacted	Transmigrated
Prevalence	18‰	2‰

Prevalence: (n cases/n patients in study) × 1000. Impacted permanent mandibular canines: (38/2119) × 1000. Transmigrated permanent mandibular canines: (5/2119) × 1000.

**Table 3:** Group A (n = 1479).

	Bilateral	Unilateral	Retained Deciduous Canines	M/F (Male/Female Ratio)	Transposition in the Lateral Incisor Region
Impacted (n = 25)	12	13	16	14/11	3
Transmigrated (n = 4)	-	4	4	1/3	-

**Table 4:** Prevalence of Impacted and Transmigrated permanent canines in the Cohort Study (n = 1479).

	Impacted	Transmigrated
Prevalence	17‰	3‰

Prevalence: (n cases/n patients in study) × 1000. Impacted permanent mandibular canines: (25/1479) × 1000. Transmigrated permanent mandibular canines: (4/1479) × 1000.

**Table 5:** Group B (n = 640).

	Bilateral	Unilateral	Retained Deciduous Canines	M/F (Male/Female Ratio)	Transposition in the Lateral Incisor Region
Impacted (n = 13)	1	12	4	8/5	2
Transmigrated (n = 1)	-	1	1	0/1	-

**Table 6:** Incidence of Impacted and Transmigrated permanent canines in the Cohort Study (n = 640).

	Impacted	Transmigrated
Incidence	20‰	1.5‰

Incidence: (n cases/n patients in study) × 1000. Impacted permanent mandibular canines: (13/640) × 1000. Transmigrated permanent mandibular canines: (1/640) × 1000.

## Data Availability

The data presented in this study are available on request from the corresponding author.
